# Expression of *DGAT2* Gene and Its Associations With Intramuscular Fat Content and Breast Muscle Fiber Characteristics in Domestic Pigeons (*Columba livia*)

**DOI:** 10.3389/fvets.2022.847363

**Published:** 2022-05-31

**Authors:** Haiguang Mao, Zhaozheng Yin, Mengting Wang, Wenwen Zhang, Sayed Haidar Abbas Raza, Fayez Althobaiti, Lili Qi, Jinbo Wang

**Affiliations:** ^1^School of Biological and Chemical Engineering, NingboTech University, Ningbo, China; ^2^College of Animal Science, Zhejiang University, Hangzhou, China; ^3^College of Animal Science and Technology, Northwest A&F University, Xianyang, China; ^4^Department of Biotechnology, College of Science, Taif University, Taif, Saudi Arabia

**Keywords:** *DGAT2*, gene expression, intramuscular fat, muscle fiber, pigeon

## Abstract

Diacylglycerol acyltransferase 2 (DGAT2) catalyzes the final step in triglyceride synthesis and plays an important role in the synthesis of fat, but the effects of its expression on intramuscular fat (IMF) content and muscle development are still unknown. In this study, we investigated the expression of the *DGAT2* gene and its associations with IMF content and breast muscle fiber characteristics in pigeons. The spatiotemporal expression profile of the pigeon *DGAT2* gene in breast muscle showed that the mRNA expression level of *DGAT2* gene in subcutaneous fat was the highest (*p* < 0.01) among eight tissues from 0 to 4 weeks of age, and showed an upward trend week by week, followed by liver (*p* < 0.05). Moreover, both mRNA and protein levels of the *DGAT2* gene in breast muscle showed an upward trend from 0 to 4 weeks (*p* < 0.05), accompanied by the upregulation of *MYOD1* and *MSTN*. In addition, the paraffin section analysis results revealed that the diameter and cross-sectional area of pectoralis muscle fiber significantly increased with age (*p* < 0.05), and a significant positive correlation was shown between the *DGAT2* gene expression level and muscle fiber diameter (*p* < 0.05). Furthermore, correlation analysis suggested that the mRNA expression level of the pigeon *DGAT2* gene was significantly (*p* < 0.01) correlated with IMF content in breast muscle. These results imply that the *DGAT2* gene has a close relationship with IMF content and breast muscle fiber characteristics in pigeons, indicating that the DGAT2 gene might be used as a candidate gene marker-assisted breeding in pigeons.

## Introduction

Intramuscular fat (IMF) content is a crucial factor in meat quality traits and is economically important in poultry breeding programs (1). With improving living standards, the demands for poultry meat are not only concentrated on carcass composition but also meat quality (2), especially in China, where people prefer to cook poultry meat by steaming and souping. Domestic pigeons are wildly raised in southern China as a kind of meat-type poultry for the abundant nutrients and delicious taste (3). Numerous studies have been carried out on other poultry, such as chickens and ducks, to identify genes regulating IMF content and muscle fiber characteristics (4, 5), but studies on domestic pigeons are still limited.

Intramuscular fat content is influenced by multiple factors, such as genetic factors, nutritional conditions, feeding method and environment, gender factors, and muscle fiber characteristics (6). It is well-known that IMF is one of the most important factors in meat quality (7). Not only does it greatly improve the meat texture and make it tenderer, but it also enhances the meat flavor and juiciness because the IMF contains a variety of flavor compounds (8). Muscle fiber characteristics can also significantly affect meat quality, mainly such as muscle fiber type, muscle fiber diameter, muscle fiber area, muscle fiber density, muscle fiber length, connective tissue, and IMF content (9, 10). IMF is the fat deposited on the perimysium, endomysium, and epimysium, so to a certain extent, the thickness of the perimysium, endomysium, and epimysium can also reflect the content of IMF (11, 12).

Many studies have demonstrated that there are many genes regulating IMF deposition and muscle fiber characteristics in meat-type animals (1, 3, 13). Diacylglycerol acyltransferase 2 (DGAT2), one of the DGAT family members, is involved in the final and rate-limiting step in the reaction of triacylglycerol synthesis pathways, consequently playing a key role in the fat deposition (1, 14). Previous studies have shown that the expression level of the *DGAT2* gene is positively correlated with IMF content in *longissimus dorsi* muscles of pigs (15) and Korean steers (16). Moreover, the *DGAT2* gene polymorphisms have been reported to affect the milk quality traits and carcass muscle in numerous animals, such as milk yield and butterfat content in goats (17), and lean percentage and backfat thickness in pigs (18, 19). More importantly, our previous research found that the variation in the *DGAT2* gene was closely associated with carcass weight, shear force, and IMF in domestic pigeons (14). Therefore, we speculated that the *DGAT2* gene was a candidate gene affecting meat quality traits in domestic pigeons. However, there have been no reports on the association of *DGAT2* gene expression with meat quality and muscle fiber characteristics traits in domestic pigeons.

Based on the above considerations, the objectives of the present study were to detect the effect of *DGAT2* gene expression on IMF content and breast muscle fiber characteristics in domestic pigeons, thereby providing a theoretical basis for the application of *DGAT2* in molecular-assisted breeding of superior pigeons.

## Materials and Methods

This research was performed according to the Chinese guidelines for animal welfare and approved by the animal welfare committee of the College of Animal Sciences, Zhejiang University (No.14814).

### Animals and Sample Collection

All pigeons used in the present study were obtained from Weitekai Pigeon Industry Co. Ltd (Wuxi, Jiangsu, China). Ten squabs of Taishen King Pigeons were randomly selected each week from the hatching day (0 weeks) to 4 weeks of age (4 weeks) to collect the breast muscle samples. In addition, 8 types of tissues were collected, such as heart, subcutaneous fat, lung, kidney, breast muscle, gizzard, proventriculus, and liver, from each pigeon. Their parents were housed in one pair (male-female paired) per cage under the same managerial conditions in a windowed poultry house and were fed a mixed-grain diet of cereals and pulses (169 g crude protein/kg and energy content of 11.47 MJ/kg), and one poultry house contained 4,000 pairs of pigeons. The left breast muscle parallel to fiber direction was sampled (1 cm × 0.5 cm × 0.5 cm), and fixed in 4% paraformaldehyde to make a microscopical section. The right breast muscle was cut into small pieces and flash-frozen in liquid nitrogen and stored at −80°C to extract RAN and protein. In addition, another set of 50 pigeons was sampled to collect the breast muscle and measure the IMF (IMF) content at 28 days of age. The IMF content was determined by the ether extraction method as has been established by the Association of Official Analytical Chemists regulations (20), and the IMF content was expressed as g of lipid in 100 g of muscle tissue.

### Total RNA Extraction, cDNA Synthesis, and Quantitative Real-Time PCR

The total RNA was extracted following the instruction book of TRlzol (Invitrogen, USA). The RNA concentration and purity were measured by the NanoDropND2000 spectrophotometer (Thermo Fisher Scientific, USA). The reverse transcription kit (Takara, China) was used to synthesize the cDNA at 42°C for 60 min with the oligo dT-adaptor primer.

The sequences of designed primer pairs are shown in Table 1. The primer pairs of *DGAT2* were designed by Primer-BLAST (https://www.ncbi.nlm.nih.gov/tools/primer-blast/) according to the pigeon *DGAT2* mRNA sequences, and then the specificity of the primers was also performed in Primer-BLAST by Primer Pair Specificity Checking. The other primers were from the previous study as shown in Table 1.

**Table 1:** Primer sequences for quantitative real-time PCR (qRT-PCR).

**Gene name**	**Primer sequences (5^**′**^ → 3^**′**^)**	**Accession number**	**Source**
*DGAT2*	F: AACGGTCCCGCAGAGATTTT	NW_004973235.1	Self-designed
	R: GTCAGTGGGTAGCGACAACA		
*MYOD1*	F: AACTGCTCTGACGGCATGAT	NW_004973198.1	(21)
	R: GTGCTTTGGATCGTTCGGTG		
*MSTN*	F: AACGGTCCCGCAGAGATTTT	NW_004973256.1	(22)
	R: GTCAGTGGGTAGCGACAACA		
*β-actin*	F: GTGGATCAGCAAGCAGGAGT	XM_005504502.2	(22)
	R: TCATCACAAGGGTGTGGGTG		

*DGAT2, diacylgycerol acyltransferase 2; MYOD1, myogenic differentiation 1; MSTN, myostatin*.

The quantitative real-time PCR (qRT-PCR) was performed by the StepOnePlus Real-Time PCR System (Applied Biosystems, USA), with a 20-μl volume, containing 2 μl cDNA (concentration 50 ng /μL), 0.8 μl forward primer (10 μmol/L), 0.8 μl reverse primer (10 μmol/L), 0.4 μl ROX Reference Dye (50×), 10 μl 2 × SYBR Premix Ex Taq II, and 6 μl nuclease-free water. The program for qRT-PCR was one cycle at 95°C for 30 s; 40 cycles at 95°C for 5 s and 60°C for 30 s. For relative quantitative, the results were normalized with the β*-actin* gene by the 2^−ΔΔCt^ method.

***Western blot*:** The protein of the breast muscle (50 mg) was extracted by the Radio Immunoprecipitation Assay (RIPA) lysis buffer with protease inhibitor. The fresh muscle tissue was washed 3 times with precooling phosphate-buffered saline (PBS) of 4°C. Then, a filter paper was used to absorb the rest of the liquid on the muscle tissue surface and then cut the tissue into several smaller pieces. Add all of the muscle tissue pieces into RIPA buffer (Beyotime, Shanghai, China) in a ratio of tissue weight (g): lysate (ml) = 1:10, and homogenized using a homogenizer until no obvious tissue mass can be seen, and then incubated on ice for 30 min (23). The tissue lysates were centrifuged at 14,000 × g at 4°C for 10 min, and the protein concentration was measured by the BCA method (BCA Protein Assay Kit, abcam, ab102536) (24). The equivalent amount (30 μg) of protein samples was mixed with a 4 × protein SDS-PAGE loading buffer (Takara, code No.9173), and the volume ratio of the loading buffer and the protein sample was 1:3. The composition of 4× protein SDS-PAGE loading buffer (1 ml) was 40 mM Tris-HCl pH8.0, 200 mM DTT, 4% SDS, 40% glycerol, and 0.032% bromophenol blue. The mixed samples were then separated by 10% SDS–PAGE gel, which is a precast polyacrylamide gel (Genscript Biotechnology Co., LTD, China, Item No. M01010C). The running buffer is the Tris-MOPS-SDS Running Buffer (Genscript Biotechnology Co., LTD, China, Item No. M00138), and the concentration is 50 mM Tris Base, 50 mM MOPS, 0.1% SDS, and 1 mM EDTA. In addition, electrophoretic at 140 V for 50 min until blue bands of bromophenol arrive at the bottom of the gel. The proteins in the gel were transferred to the polyvinylidene difluoride (PVDF) membranes (Millipore), which were blocked with 5% BSA in Tris-buffered saline containing 0.1% Tween-20 (TBST) at room temperature for 2 h. The transferring was performed by the Bio-Rad standard wet membrane transfer unit under ice bath conditions with 300 mA transferring electric current and 60–70 V transferring voltage, and the transferring time is 50 min. The membrane was then incubated overnight at 4°C with the primary antibodies (Anti-DGAT2, abcam, ab237613) (1:1000 dilution). After that, the membranes were incubated with the secondary antibody (Goat anti-rabbit IgG-HRP, absin, abs2002) for 2 h at room temperature with a secondary antibody concentration of 0.2 μg/ml. The immunoreactive bands were visualized by the Developer and Fixer Kit (BEYOTIME, Shanghai, China), and images were captured by the chemiluminescent imaging system (Sagecreation, China). The densities of the bands were quantified by the NIH Image J software. The ratios of the proteins to the reference proteins (GAPDH, abcam, ab8245) were used as the relative quantitative analysis results.

### Histological Analysis of the Breast Muscle

The fixed breast muscle samples were embedded into the paraffin, 5 μm thick of serial cross-sections perpendicular to the direction of muscle fibers were made in the cryostat at −20°C and then stained with the hematoxylin and eosin (H&E). The Pannoramic Viewer software was applied to conduct the histological analysis, including the muscle fiber diameter (D), cross-sectional area (A), and density (d).

Measurement for the muscle fiber cross-sectional area (A): at least 240 breast muscle fibers were measured in six fields randomly under the 40-fold objective microscope. The mean values were represented as the muscle fiber cross-sectional area.

Measurement for muscle fiber diameter (D): on the assumption that the muscle fibers were round, the muscle fiber diameter was calculated by the following formula, D = 2 √ (A/π). The mean values were represented as the muscle fiber diameter.

Measurement for the muscle fiber density: the total area (S) and the number of muscle fibers (N) were calculated by the image analysis software in six different fields of vision, and the density (d) of muscle fibers was calculated by the following, d = N/S. The mean values were represented as the muscle fiber diameter.

At the age of 4 weeks, oil red O staining was used on frozen sections of pigeon breast muscle. The slide was incubated in propylene glycol for 2 min and then incubated in oil red O solution for about 6 min. Then the sections were differentiated in the 85% propylene glycol for 1 min and rinsed two times in clear water. After that, the sections were incubated in hematoxylin for 2 min and then rinsed 3 times in clear water, and finally coverslips with an aqueous mounting medium were placed on top of the sections. The sections were examined under a microscope for imaging to observe the amount and size distribution of intramuscular adipocytes.

### Statistical Analysis

Statistical analysis was performed by the SPSS20.0 (SPSS, Chicago, IL). Differences between groups were analyzed by one-way ANOVA followed by Bonferroni contrast adjusted for multiple comparisons. The date were presented as mean ± SE. The value of *p* < 0.05 represents statistically significant and *p* < 0.01 represents highly significant. The correlation analysis was performed by bivariate correlation with Pearson's correlation.

## Results

**Figure 1:**
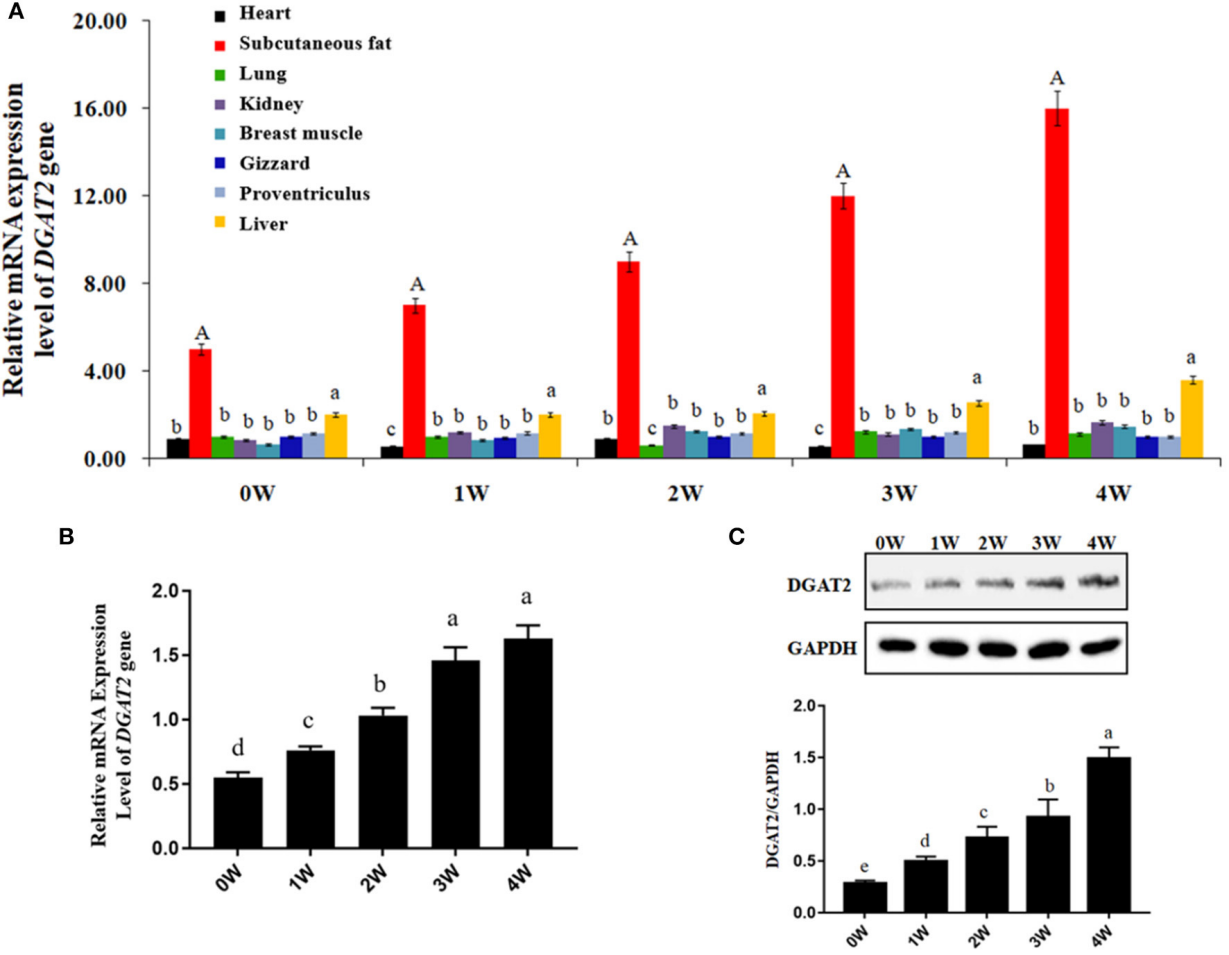
Gene and protein expression levels. **(A)** Absolute quantification of mRNA expression levels of *diacylgycerol acyltransferase 2 (DGAT2)* gene in different tissues at 0, 1, 2, 3, and 4 weeks post-hatching in domestic pigeons. Bars with capital letter and lowercase letter mean highly significant differences (*p* < 0.01) and significant differences (*p* < 0.05), respectively. Data were represented as mean ± SE for 10 pigeons (*n* = 10). **(B)** Relative mRNA expression levels *of DGAT2* gene in the breast muscle of domestic pigeons at 0, 1, 2, 3, and 4 weeks post-hatching. Bars with lowercase letters meant significant differences (*p* < 0.05). Data were represented as mean ± SE for 10 pigeons (*n* = 10). Group 0 week was used as reference. **(C)** Western blot analysis of DGAT2 protein in the breast muscle of domestic pigeons at 0, 1, 2, 3, and 4 weeks post-hatching, normalized with GAPDH. Bars with different lowercase letters were significant differences (*p* < 0.05).

The absolute quantitative mRNA expression levels of the *DGAT2* gene in 8 tissues (heart, subcutaneous fat, lung, kidney, breast muscle, gizzard, proventriculus, and liver) at 0, 1, 2, 3, and 4 weeks post-hatching in domestic pigeons were shown in Figure 1A. The results showed that the mRNA expression level of the *DGAT2* gene in subcutaneous fat showed the highest (*p* < 0.01) among eight tissues of all the ages and showed an upward trend week by week, followed by the liver (*p* < 0.05). The mRNA expression levels in the heart, lung, kidney, breast muscle, gizzard, and proventriculus were relatively low in all the detected weeks.

The results of relative mRNA expression levels of the *DGAT2* gene in the breast muscle of domestic pigeons from 0 to 4 weeks of age post-hatching are shown in Figure 1B. It was obvious that the mRNA expression levels of the *DGAT2* gene in breast muscle revealed a significant upward trend (*p* < 0.05) from 0 to 3 weeks of age, and then reached the highest level at the age of 4 weeks.

The results of western blot analysis of DGAT2 protein in the breast muscle of domestic pigeons from 0 to 4 weeks of age post-hatching are shown in Figure 1C. After hatching, the DGAT2 protein expression levels significantly increased (*p* < 0.05) by the weeks of age, and finally reached the peak level at the age of 4 weeks.

**Figure 2:**
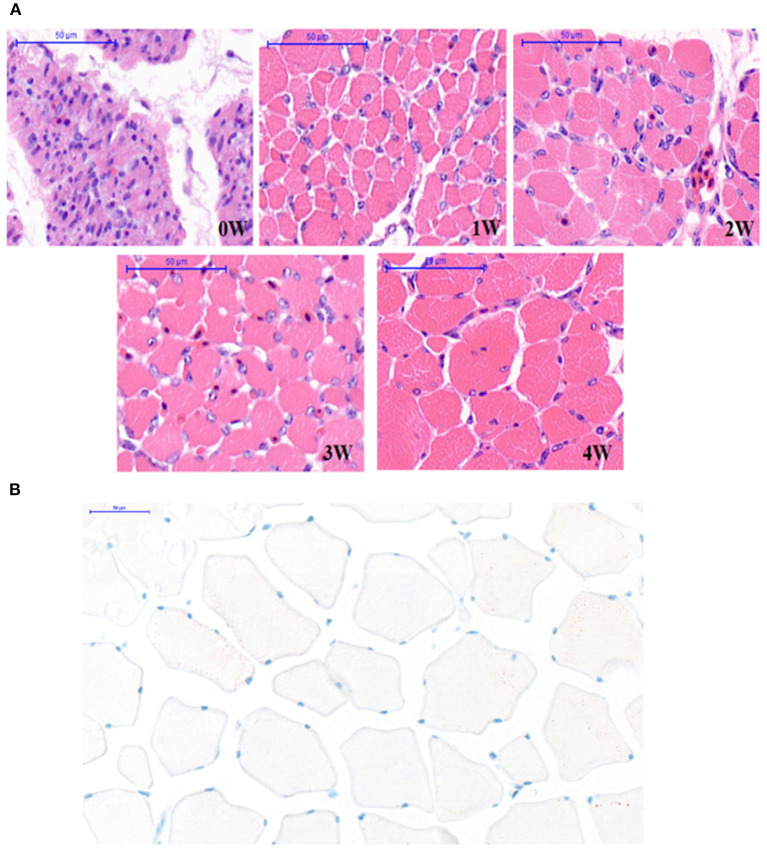
Section of pectoral muscle tissue. **(A)** The breast muscle fiber morphology of pigeons (*n* = 10) with hematoxylin-eosin (H&E) staining at 0, 1, 2, 3, and 4 weeks after hatching (40×). Scale bar: 50 μm. **(B)** Oil red O staining of frozen sections of pigeon breast muscle of 4 weeks (*n* = 50). Scale bar: 50 μm.

The representative microphotographs of breast muscle fibers from 0 to 4 weeks of age are shown in Figure 2A. At 0 weeks of age (the hatching day), it was difficult to distinguish the muscle fibers for the myoblast cells clustered together and forming multinucleated muscular tubes. At 1 week of age, individually distinguishable muscle fibers started to occur with the development of the muscular tubes, and the endomysium also began to appear clearly among the muscle fibers. Afterward, the breast muscle fibers continuously developed from 2 to 4 weeks of age, and finally became full and plump at the age of 4 weeks. In addition, a perimysium could be observed in the breast muscle during all the weeks of age. Oil red O staining was used on frozen sections of pigeon breast muscle at the age of 4 weeks (Figure 2B). The distribution of intramuscular adipocytes was displayed.

**Figure 3:**
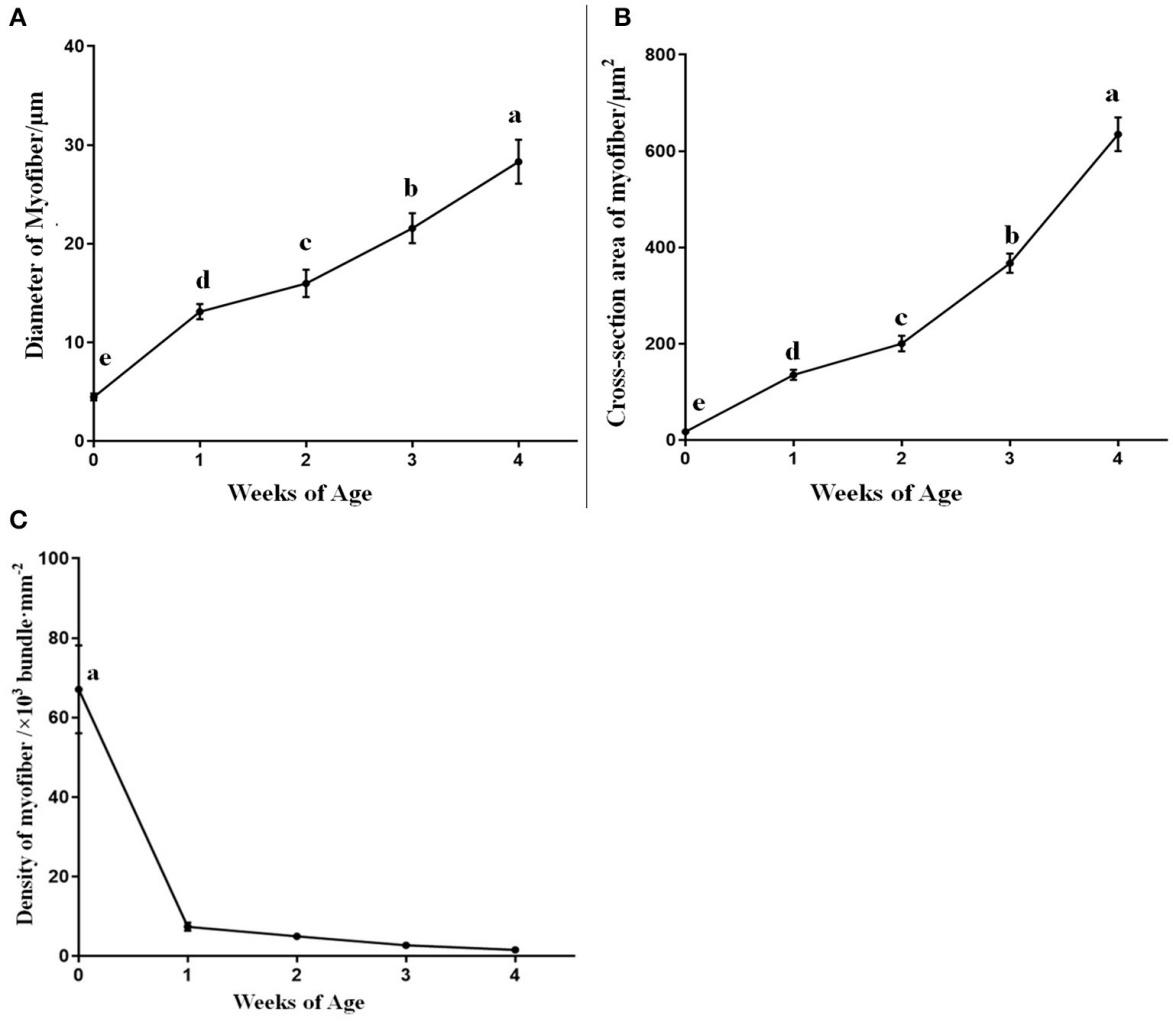
The diameter, cross-section area, and density of breast muscle fibers in domestic pigeons at 0, 1, 2, 3, and 4 weeks post-hatching. Lowercase letters mean significant differences (*p* < 0.05). Data were represented as mean ± SE for 10 pigeons (*n* = 10).

The corresponding muscle fiber diameter, cross-section area, and density are shown in Figures 3A–C. The diameter and cross-section area of breast muscle fibers significantly increased (*p* < 0.05) by the weeks of age from 0 to 4 weeks. Moreover, an enormous increase in muscle fiber diameter and cross-section area was observed from 0 to 1 week of age. On the contrary, the density of the muscle fibers significantly dropped (*p* < 0.05) from 0 to 1 week, and since then the decline continued slowly in the following weeks (*p* > 0.05).

**Figure 4:**
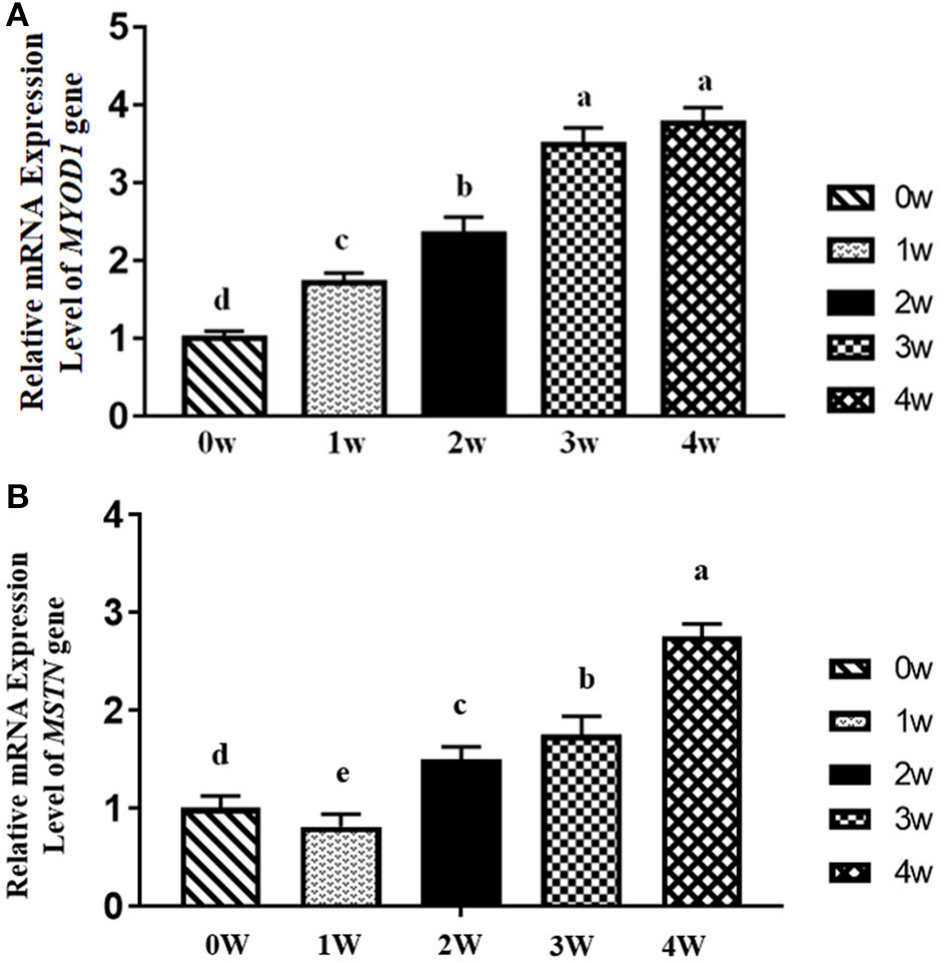
Relative mRNA expression levels *of myogenic differentiation 1 (MYOD1)* and *myostatin (MSTN)* gene in breast muscle of domestic pigeons at 0, 1, 2, 3, and 4 weeks post-hatching. β*-actin* was used as the reference gene. Bars with lowercase letters meant significant differences (*p* < 0.05). Data were represented as mean ± SE for 10 pigeons (*n* = 10). **(A)** Relative mRNA expression levels *of MYOD1*. Group 0 week was used as reference. **(B)** Relative mRNA expression levels *of MSTN*. Group 0 week was used as reference.

The results of the relative mRNA expression levels *of MYOD1* and *MSTN* genes in the breast muscle of domestic pigeons from 0 to 4 weeks of age post-hatching are shown in Figure 4. It was obvious that the mRNA expression levels of the *MYOD1* gene in breast muscle showed a significant upward trend (*p* < 0.05) from 0 to 3 weeks of age, and then reached their peak level at the age of 4 weeks, which is similar to the mRNA expression levels of *DGAT2* gene in Figure 1B. The mRNA expression levels of the *MSTN* gene showed a significant decrease (*p* < 0.05) in the first-week post-hatching, and then continued to increase in the next few weeks, and finally reaching the highest level at the age of 4 weeks.

The correlation coefficients (*r*) of the mRNA and protein expression levels of the *DGAT2* gene with breast muscle fiber diameter, cross-section area, and density at 4 weeks of age are shown in Table 2. The results revealed significant positive correlations between the mRNA expression levels of the *DGAT2* gene and fiber diameter (*r* = 0.8851, *p* < 0.05) as well as cross-section area (*r* = 0.9420, *p* < 0.01), and the DGAT2 protein expression level also showed a significant positive correlation with the muscle fiber diameter (*r* = 0.8041, *p* < 0.05) and cross-section area (*r* = 0.9062, *p* < 0.01) of the myofiber. In addition, a significant negative correlation was revealed between the mRNA expression level of the *DGAT2* gene and the density of breast muscle fibers (*r* = −0.7190, *p* < 0.05), and the same significant negative correlation was noticed on the DGAT2 protein level (*r* = −0.5474, *p* < 0.05). Correlation coefficients (*r*) between the relative mRNA expression level of the pigeon *DGAT2* gene and IMF content in breast muscle at 4 weeks of age in pigeons are also shown in Table 2. The result indicated that the mRNA expression level of the pigeon *DGAT2* gene was significantly (*r* = 0.7720, *p* < 0.01) correlated with IMF content in breast muscle, and the DGAT2 protein expression also showed a significant difference with the IMF content in breast muscle (*r* = 0.6235, *p* < 0.05). In addition, correlation coefficients (*r*) analysis between both relative mRNA (*r* = 0.5246, *p* < 0.05) and protein expression level (*r* = 0.4852, *p* < 0.05) of pigeon *DGAT2* and the number of intramuscular adipocytes showed a significant difference, while there was no difference with the size of intramuscular adipocytes (*p* > 0.05).

**Table 2:** Correlation coefficients (*r*) between the mRNA/protein expression level of *DGAT2* gene in breast muscle and muscle fiber diameter, cross-section area and density, intramuscular fat (IMF) content, and amount and size of intramuscular adipocytes of 4 weeks of age in pigeons.

**Measurements**	**The mRNA expression of *DGAT2* gene**	**The protein expression of DGAT2**
Diameter of myofiber	0.8851*	0.8041*
Cross-section area of myofiber	0.9420**	0.9062**
Density of myofiber	−0.7190*	−0.5474*
IMF content	0.7720**	0.6235*
Amount of intramuscular adipocytes	0.5426*	0.4852*
Size of intramuscular adipocytes	0.2135	0.2083

Values with ** and * meant highly significant differences (p < 0.01) and significant differences (p < 0.05), respectively.

## Discussion

Diacylglycerol acyltransferase 2 is a key member of the DGAT family and plays an important role in the synthesis of fat (1, 14). It is well-known that DGAT2 is essential for the fundamental synthesis of triglycerides, for it is involved in the final and rate-limiting step in the reaction of triacylglycerol synthesis pathways (17–19). Therefore, many previous studies have considered it as an important candidate gene for meat quality and carcass traits in meat-type animals (14, 19). Meanwhile, it is also regarded as a candidate gene for milk fat percentage in milk-type animals mainly focused on ruminants (25–27). Many studies have reported that the expression of *DGAT2* is significantly associated with meat quality traits and carcass traits, especially on IMF content in pigs, steers, yaks, and chickens (1, 2, 15, 16). It is worth noting that in our previous study, we found that *DGAT2* gene polymorphisms were significantly associated with IMF content and muscle tenderness in domestic pigeons, indicating that *DGAT2* might be a promising candidate gene for marker-assisted breeding in pigeons (14). However, there is no study that has reported on the effect of *DGAT2* gene expression on meat quality and muscle fiber characteristics traits in domestic pigeons. Therefore, in the present study, we investigated the associations between the expression of the *DGAT2* gene and IMF content, as well as breast muscle fiber characteristics in domestic pigeons.

We first established a spatiotemporal expression profile of the *DGAT2* gene in eight tissues, namely, the heart, subcutaneous fat, lung, kidney, breast muscle, gizzard, proventriculus, and the liver, from birth (0 weeks) to 4 weeks of age (4 weeks). As we expected, *DGAT2* is widely expressed in all the tested tissues, and subcutaneous fat showed the highest (*p* < 0.01) expression level among all the eight tissues at all weeks of age, showing an upward trend week by week, followed by liver (*p* < 0.05). This result is consistent with previous studies reported on yaks, sheep, and chickens (1, 2, 28). DGAT2 is expressed widely in a variety of tissues in animals (1, 29, 30). The high expression level of *DGAT2* in fat tissue indicates an important function in the synthesis and storage of triglycerides (31, 32). In addition, the mRNA expression levels and protein expression levels of DGAT2 in breast muscle revealed a significant upward trend from 0 to 3 weeks of age and then reached the highest level at the age of 4 weeks. As the individual grows and develops, the IMF content continues to deposit and increase within a certain week of age (33), which is consistent with the expression trend of the *DGAT2* gene in the breast muscle. Moreover, the mRNA expression level of the pigeon *DGAT2* gene was significantly (*p* < 0.01) correlated with IMF content in breast muscle (*R* = 0.7720). This also explains well that the *DGAT2* gene plays an important role in IMF deposition. It is interesting to note that the liver also showed a high expression level of the DGAT2 gene because the liver plays an important role in lipid digestion, absorption, synthesis, decomposition, and transportation (34). It also proved the importance of DGAT2 in fat synthesis from another aspect.

Intramuscular fat content is one of the most important acknowledged factors in meat quality (35). Not only does it greatly improve the meat tenderness and texture (10, 11), but it also enhances the meat flavor and juiciness for the IMF as it contains a variety of flavor compounds (8, 36). IMF content is influenced by multiple factors, such as genetic background, gender, nutritional condition, feeding method, and environment and muscle fiber characteristics (6). Muscle fiber characteristics, mainly including muscle fiber type, muscle fiber diameter, muscle fiber area, muscle fiber density, muscle fiber length, connective tissue, and IMF content, can significantly affect meat quality (9, 10). IMF is the fat deposited on the perimysium, endomysium, and epimysium, so to a certain extent, the thickness of the perimysium, endomysium, and epimysium can also reflect the content of IMF (11, 12). Therefore, muscle fiber characteristics could significantly influence meat quality. In this study, we found significant positive correlations between the mRNA expression levels of the *DGAT2* gene and fiber diameter (*p* < 0.05) as well as cross-section area (*p* < 0.01), and the DGAT2 protein expression level also showed a significant positive correlation with the cross-section area (*p* < 0.01) of the myofiber. MYOD1 and MSTN are the two acknowledged gene-affected muscle fiber characteristics (21, 22). The *MYOD1* plays an important role in muscle development and growth from commitment and proliferation through the formation of muscle fibers (37, 38). The MSTN is a negative regulator of skeletal muscle growth and plays an important role in muscle development (22). The present study showed that *the DGAT2* gene in breast muscle revealed a significant upward trend with the ages of pigeons, coupling with the changes in both genes (*MSTN* and *MYOD1*). Furthermore, a significant negative correlation was revealed between the mRNA expression level of the *DGAT2* gene and the density of breast muscle fibers, and the same significant negative correlation was noticed on the DGAT2 protein level. Interestingly, *DGAT2* was positively correlated with muscle fiber diameter, but it was also correlated with IMF content in the breast muscle of pigeons. A previous study had shown that the DGAT2 gene was not only involved in fat synthesis and deposition but also influenced muscle growth and carcass composition (14–16). Above all, DGAT2 might influence IMF content by affecting the muscle fiber development in pigeons.

In conclusion, our results in this study showed that the *DGAT2* gene might play an important role in the meat quality of pigeons by affecting the IMF deposition and breast muscle fiber development, indicating that the *DGAT2* gene might be used as a candidate gene marker-assisted breeding in pigeons. Further functional research should be carried out in the following study to confirm our conclusions.

## Data Availability Statement

The original contributions presented in the study are included in the article/supplementary material, further inquiries can be directed to the corresponding author/s.

## Ethics Statement

The animal study was reviewed and approved by the animal welfare committee of the College of Animal Sciences, Zhejiang University.

## Author Contributions

HM analyzed the data and drafted the manuscript. ZY provided experiment suggestions for this study. MW and WZ performed the RNA extraction and qRT-PCR. SR and FA provided constructive suggestions for the discussion and validation revised manuscript critically for writing—review and editing. LQ and JW conceived the project and designed the experiments. All authors have read and agreed to the published version of the manuscript.

## Funding

The research was supported by the Key Agriculture Science and Technology Project (No. 2016C02054-16) in Zhejiang province of China, the Ningbo Science and Technology Bureau Project (No. 202003N4305), the Talent Introduction Fund of NingboTech University (20211018Z0216), and the Taif University Research Supporting Project number (TURSP-2020/222), Taif University, Taif, Saudi Arabia.

## Conflict of Interest

The authors declare that the research was conducted in the absence of any commercial or financial relationships that could be construed as a potential conflict of interest.

## Publisher's Note

All claims expressed in this article are solely those of the authors and do not necessarily represent those of their affiliated organizations, or those of the publisher, the editors and the reviewers. Any product that may be evaluated in this article, or claim that may be made by its manufacturer, is not guaranteed or endorsed by the publisher.
