# Corrigendum: Expression of *DGAT2* gene and its associations with intramuscular fat content and breast muscle fiber characteristics in domestic pigeons (*Columba livia*)

**DOI:** 10.3389/fvets.2022.1028657

**Published:** 2022-09-15

**Authors:** Haiguang Mao, Zhaozheng Yin, Mengting Wang, Wenwen Zhang, Sayed Haidar Abbas Raza, Fayez Althobaiti, Lili Qi, Jinbo Wang

**Affiliations:** ^1^School of Biological and Chemical Engineering, NingboTech University, Ningbo, China; ^2^College of Animal Science, Zhejiang University, Hangzhou, China; ^3^College of Animal Science and Technology, Northwest A&F University, Xianyang, China; ^4^Department of Biotechnology, College of Science, Taif University, Taif, Saudi Arabia

**Keywords:** *DGAT2*, gene expression, intramuscular fat, muscle fiber, pigeon

In the published article, there was an error in “affiliation 1.” Instead of “School of Biological and Chemical Engineering, Ningbo Tech University, Ningbo, China,” it should be “School of Biological and Chemical Engineering, NingboTech University, Ningbo, China.”

The authors apologize for this error and state that this does not change the scientific conclusions of the article in any way. The original article has been updated.

## Publisher's note

All claims expressed in this article are solely those of the authors and do not necessarily represent those of their affiliated organizations, or those of the publisher, the editors and the reviewers. Any product that may be evaluated in this article, or claim that may be made by its manufacturer, is not guaranteed or endorsed by the publisher.

